# Investigating the Role of Serotonin in Methamphetamine Psychosis: Unaltered Behavioral Effects of Chronic Methamphetamine in 5-HT_1A_ Knockout Mice

**DOI:** 10.3389/fpsyt.2017.00061

**Published:** 2017-04-20

**Authors:** Emily J. Jaehne, Dzeneta Ameti, Tehani Paiva, Maarten van den Buuse

**Affiliations:** ^1^Department Psychology and Counselling, School of Psychology and Public Health, La Trobe University, Melbourne, VIC, Australia; ^2^Department of Pharmacology, University of Melbourne, Melbourne, VIC, Australia; ^3^The College of Public Health, Medical and Veterinary Sciences, James Cook University, Townsville, QLD, Australia

**Keywords:** methamphetamine, psychosis, serotonin, 5-HT_1A_ receptors, neuroplasticity

## Abstract

Methamphetamine (Meth) is a widely abused stimulant drug, but this abuse is associated with an increased risk of developing psychosis. In addition to its well-known action on brain dopamine, Meth also affects serotonergic (5-HT) neurons. The aim of this study was to investigate this role in mice, which lack one of the main serotonin receptors, the 5-HT_1A_ receptor, which has been implicated in both schizophrenia and Meth-induced psychosis. Male and female wild-type or 5-HT_1A_ knockout (KO) mice received daily treatment with increasing doses of methamphetamine from 6 to 9 weeks of age (1–4 mg/kg/day twice a day). At least 2 weeks after the last injection, the mice underwent a battery of behavioral tests focusing on psychosis-related behaviors, including Meth-induced hyperactivity, prepulse inhibition (PPI), social interaction, elevated plus maze (EPM), and Y-maze. Meth pretreatment resulted in significantly increased hyperlocomotion in response to an acute Meth challenge, but this effect was independent of genotype. Chronic Meth treatment resulted in decreased levels of anxiety in the EPM in both sexes, as well as increased startle responses in female mice only, again independent of genotype. 5-HT_1A_ KO mice showed an increased locomotor response to acute Meth in both sexes, as well as increased PPI and decreased startle responses in female mice only, independent of Meth pretreatment. In conclusion, the effects of chronic Meth appear unaffected by the absence of the 5-HT_1A_ receptor. These results do not support a role of the 5-HT_1A_ receptor in Meth-induced psychosis.

## Introduction

Methamphetamine (Meth) use and availability has been increasing worldwide and is placing a huge burden on users, their relatives and friends, and society at large (1). One of the most severe consequences of Meth abuse in some users is the development of psychosis (2, 3), either acutely when the drug is active or as long-term symptoms, which may be similar to schizophrenia (2–4). Users may also develop other persistent psychiatric symptoms or impaired cognitive abilities following prolonged use of the drug (4, 5). However, the mechanisms involved in Meth psychosis, and its overlap with schizophrenia symptoms, remain unclear.

We have previously investigated the effects of chronic Meth on psychosis-like behavior in adulthood using a dosing schedule of escalating exposure during late adolescence/early adulthood. At least 2 weeks after the chronic treatment ended, the long-lasting effects of chronic Meth on a locomotor hyperactivity model of psychosis, as well as on cognition and other behaviors with relevance to psychiatric symptoms, were studied (6–8). Acute amphetamine- or Meth-induced locomotor hyperactivity is a widely used behavioral test in preclinical schizophrenia research, as it models the increase in dopamine signaling thought to contribute to psychosis (9). Sensitization of this dopaminergic signaling has been postulated to mimic developmental mechanisms in psychosis (10, 11). In our experiments, pretreatment with Meth led to sensitization to the acute effects of amphetamine in the hyperactivity model compared to control pretreatment (6), while Meth pretreatment reduced the effect of an acute injection of amphetamine on prepulse inhibition (PPI) of acoustic startle (PPI) (7). Further studies showed that the chronic dosing protocol altered social novelty behavior but not short-term memory in the Y-maze (8).

There is increasing evidence for a role of serotonin, as well as dopaminergic mechanisms, in schizophrenia. Postmortem studies have shown increased expression of 5-HT_1A_ receptors in the frontal cortex (12) but decreased binding in the amygdala in patients with schizophrenia (13). A polymorphism in the 5-HT_1A_ receptor was associated with schizophrenia psychopathology (14) and several atypical antipsychotic drugs have high affinity for serotonergic receptors, including the 5-HT_1A_ receptor (15–19). Extensive evidence suggests that 5-HT_1A_ receptor activation modulates dopamine activity and may enhance cognition in schizophrenia (15, 17, 20, 21). While much of the animal literature has focused on the role of 5-HT_1A_ in anxiety, where studies have shown 5-HT_1A_ knockout (KO) mice to have a robust anxiety phenotype behavior in the elevated plus maze (EPM) (22–25) and certain fear conditioning paradigms (26), more recent work has investigated its role in schizophrenia-related behaviors. For example, 5-HT_1A_ KO mice have an enhanced locomotor response to d-amphetamine compared to wild-type (WT) mice (27), an altered PPI response to methylenedioxymethamphetamine (28), and impaired cognition in the Morris Water maze (25, 29). The 5-HT_1A_ antagonist WAY100635 has also been shown to increase PPI in C57BL/6 mice (30).

These previous studies suggest a role for 5-HT_1A_ receptors in schizophrenia although it is less clear if this extends to Meth psychosis. While several studies have suggested a role for 5-HT_1A_ receptors in addiction to this drug (31), a role for this receptor in sensitization of Meth-induced hyperlocomotion is less clear (32). For example, the 5-HT_1A_ receptor antagonist, WAY100635, had no effect on amphetamine or Meth-induced hyperlocomotor activity (33, 34), while in contrast, the agonist, 8-OH-DPAT, was able to inhibit hyperlocomotion (33). 8-OH-DPAT and the 5-HT_1A_ receptor agonist, osemozotan, also prevented development and expression of amphetamine or Meth-induced behavioral sensitization (35, 36). However, the long-lasting effects of chronic Meth to induce psychosis-like behavior have not been studied in animals with genetically modified 5-HT_1A_ receptor levels. The current study was, therefore, designed to investigate the effect of 5-HT_1A_ receptor KO on the action of Meth to induce psychosis- and schizophrenia-related behaviors. We used the acute Meth-induced hyperactivity model of psychosis as well as a range of other relevant behavioral tests, including PPI for sensorimotor gating, social interaction as a model of some of the negative symptoms of schizophrenia, Y-maze and fear conditioning to assess cognitive changes, and EPM for anxiety. 5-HT_1A_ KO mice or WT controls were tested during adulthood after a 3-week binge protocol of Meth administration during late adolescence. As previous studies have shown sex differences in animal models of psychosis (37) as well as the effect of this Meth dosing protocol on some behaviors (7, 8), both male and female mice were included in this study.

## Materials and Methods

### Animals

5-HT_1A_ receptor KO mice and their C57BL/6 WT control littermates (WT) (27) were derived from a breeding colony at the La Trobe Animal Research and Teaching Facility. Heterozygous mice were used as breeders to obtain WT and KO littermates for the current studies, while heterozygous offspring was not used. Genotypes were confirmed at weaning by Transnetyx Inc. (Cordova, TN, USA).

A total of 104 male and female mice were used for experiments (*n* = 10–16/group; Table 1). All mice were housed in groups of two to five during the experimental period in individually ventilated cages (Tecniplast, Buguggiate, Italy) with standard pellet food and water available *ad libitum*. Ambient temperature of housing and testing rooms was 21 ± 2°C and mice were housed under a 12-h light–dark cycle, lights on at 0700 hours, with all behavioral testing conducted between 0800 and 1600 hours.

**Table 1:** **Number and mean weight of experimental groups**.

Group	*n*	Body weight (g) at end of testing
**Males**		
WT-Saline	12	28.0 ± 0.7
WT-Meth	12	30.4 ± 0.6
5-HT_1A_ KO-Saline	12	28.8 ± 0.7
5-HT_1A_ KO-Meth	16	29.6 ± 0.5
**Females**		
WT-Saline	12	22.4 ± 0.3
WT-Meth	13	22.3 ± 0.3
5-HT_1A_ KO-Saline	16	22.5 ± 0.4
5-HT_1A_ KO-Meth	11	23.4 ± 0.4

*Genotype: 5-HT_1A_ knockout (KO) or wild-type (WT)*.

*Pretreatment: saline or methamphetamine (Meth)*.

*Body weight: mean (±SEM)*.

### Chronic Methamphetamine Treatment and Experimental Procedure

Mice were given either Meth or saline as a vehicle control for five consecutive days a week for a period of 3 weeks during adolescence, from the age of 6 to 9 weeks. Mice received 1 injection/day of 1 mg/kg during the first week, 2 injections/day of 2 mg/kg the second week, and 2 injections/day of 4 mg/kg the third week (6–8). This protocol was based on a binge-type Meth intake pattern seen in many abusers of the drug, including occasional interruptions of administration and a gradual increase of doses (38). The 2-week washout is required to be able to see the long-term effects of these chronic doses, which reflect increased effects in relapsed chronic users (38) as well as sensitization mechanisms similar to those postulated in psychosis development (10, 11). Meth and saline solutions were given intraperitoneally at a volume of 5 ml/kg. Meth was purchased from the National Measurement Institute (Pymble, NSW, Australia) and dissolved in 0.9% sterile saline.

Starting 2 weeks following the final injection, at 11 weeks of age, mice underwent a battery of behavioral tests over a period of 4 weeks, with less stressful tests performed first and more stressful tests, or tests involving acute drug challenge, performed at the end. Mice were given at least 2–3 days between behavioral tests. Tests were performed in the following order: Y-maze, social interaction, EPM, fear conditioning, PPI, and Meth-induced locomotor hyperactivity.

### Behavioral Testing

#### Methamphetamine-Induced Locomotor Hyperactivity

Mice were placed into automated photocell arenas (Med Associates, Fairfax, VT, USA), 27 cm × 27 cm with walls 40 cm high, with a 16 × 16 array of photobeam sensors for detecting movement. Each time, they were first habituated in the arenas for 1 h, then were removed briefly and injected with the challenge drug, then placed back into the arena for a further 2 h (6, 27). During the first session, all mice received saline, followed by 1 mg/kg Meth in the second and 3 mg/kg Meth in the final session, with 3–4 days washout in between sessions. Distance traveled was automatically calculated in 5-min time bins.

#### PPI of Acoustic Startle

Prepulse inhibition was assessed as a measure of sensorimotor gating using automated SR-Lab startle chambers (San Diego Instruments, San Diego, CA, USA). Mice were placed in individual plexiglass cylinders (5 cm diameter) and the test session consisted of 104 stimulus trials as previously described (7, 27). PPI was quantified as the difference between stimulus responses during prepulse-pulse and pulse-alone trials and expressed as a percentage of pulse-alone responses. At 30 ms ISI, mice showed no effect of genotype or pretreatment, therefore, results analysis will focus on the 100 ms ISI.

#### Social Interaction

The test apparatus consists of a rectangular three chambered enclosure, 43 cm × 64 cm, with transparent walls 23 cm high. Two “stranger” enclosures, diameter 9 cm, height 10 cm, were placed in the two outer chambers. The test consisted of three phases, each 10 min in duration, which were conducted immediately after one another (8, 39). Stranger mice were adolescent 5-HT_1A_ heterozygous mice of the same sex as the test mouse. Interaction time was measured using Ethovision video tracking (Noldus, Wageningen, The Netherlands), with time spent in a “sniffing zone,” 2.5 cm immediately surrounding stranger cages, defined as interaction.

#### Elevated Plus Maze

The EPM consisted of an elevated plus-shaped platform with two open and two closed arms, with a length of 40 cm and width of 5 cm, 50 cm above the ground, with a central square section between arms (40, 41). During the 5-min trial, time spent in open and closed arms and number of entries into arms were measured using Ethovision video tracking (Noldus). Mice that spend more time in the open arms are considered to have a lesser anxiety phenotype. The total number of arm entries was used as a control measure of locomotor activity.

#### Y-Maze

The Y-maze was a Y-shaped apparatus with three arms (start arm and two test arms), each 32 cm long and 10 cm wide with walls 15 cm high. The arms were at a 120° angle from each other. The test arms had different black and white symbols on either end wall. Behavior was tracked using Ethovision (Noldus), which measured time spent in, and number of entries to, each arm (42, 43). Sessions included a 10-min trial with access to only two arms followed 1 h later but a 5 min re-trial with access to all three arms. Time spent in the novel test arm compared to the other arms (familiar and start) during the retention phase was used as a measure of short-term spatial recognition memory.

#### Fear Conditioning

Fear memory was assessed using a 3-day fear conditioning protocol as previously used in mice (43, 44) using chambers equipped with footshock grid floors (Med Associates). Two different conditioning contexts were used, which differed in lux, scent, bedding, and structure due to a concave wall insert, and mice were pseudorandomly assigned to one context or the other. Freezing was defined as complete lack of any movement besides breathing and was measured in response to (1) the context where mice had previously been exposed to an unconditioned stimulus (scrambled foot-shocks of 1 s duration, 0.7 mA) or (2) a conditioned stimulus (30 s duration, 7,500 Hz, 70 dB) previously presented prior to the US, but in a new context.

### Statistical Analysis

Data were expressed as the mean ± SEM and differences between groups were analyzed with analysis of variance (ANOVA), with repeated measures where appropriate, using IBM SPSS Statistics 23 (Armonk, New York, NY, USA). All data were first analyzed with genotype, Meth pretreatment and sex as between-group statistical factors. If significant interactions were seen, data were split and further analyzed by genotype, pretreatment, or sex as stated in the results. For locomotor activity analysis, repeated-measures factors were time and acute Meth treatment, while for social interaction and Y-maze analysis, repeated measures factors were stranger mouse interaction time and time spent in arms, respectively. Differences between groups were considered significant when *p* < 0.05.

## Results

### Locomotor Activity

**Figure 1:**
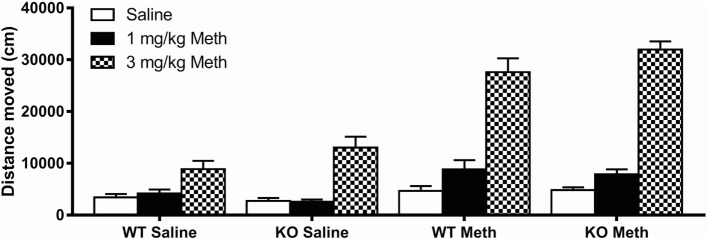
**Acute methamphetamine (Meth) induced hyperactivity following chronic Meth exposure**. A low acute challenge dose of Meth (1 mg/kg) induced hyperactivity in mice previously pretreated with Meth. There was no genotype effect at this dose. A high acute challenge dose of Meth (3 mg/kg) induced hyperactivity to a significantly greater extent in Meth pretreated compared to saline pretreated mice. This dose also induced hyperactivity to a significantly greater extent in 5-HT_1A_ knockout (KO) mice compared to wild-type (WT) mice. Results are shown for males and females combined.

Analysis of locomotor activity following 1 and 3 mg/kg Meth compared to saline control showed a significant main effect of an acute Meth challenge [*F*_(2, 188)_ = 273, *p* < 0.001] and a treatment × pretreatment interaction [*F*_(2, 188)_ = 73.3, *p* < 0.001]. Data were, therefore, split and further ANOVAs were used to compare the effect of each dose of acute Meth with saline to further explore this relationship. Although females showed higher activity than males [*F*_(1, 94)_ = 16.4, *p* < 0.001], there were no significant interactions between sex and genotype, acute treatment or pretreatment, therefore, locomotor activity results for males and females were combined (Figure 1).

Following acute injection with 3 mg/kg Meth, all groups showed a significant main effect of treatment compared to saline injection [*F*_(1, 94)_ = 307, *p* < 0.001]. This effect was much greater in Meth-pretreated mice [treatment × pretreatment *F*_(1, 94)_ = 87.3, *p* < 0.001], indicating sensitization to a challenge dose of Meth following the binge dosing protocol used, and was also greater in 5-HT_1A_ KO mice than in WT controls [treatment × genotype *F*_(1, 94)_ = 5.10, *p* = 0.026]. There was, however, no treatment × pretreatment × genotype interaction, suggesting the genotype differences and Meth sensitization were independent of each other.

Acute injection with 1 mg/kg Meth showed similar results [treatment *F*_(1, 94)_ = 24.9, *p* < 0.001; treatment × pretreatment *F*_(1, 94)_ = 18.0, *p* < 0.001]; however, the effect of genotype in response to acute Meth failed to reach significance at this dose.

### Prepulse Inhibition

**Figure 2:**
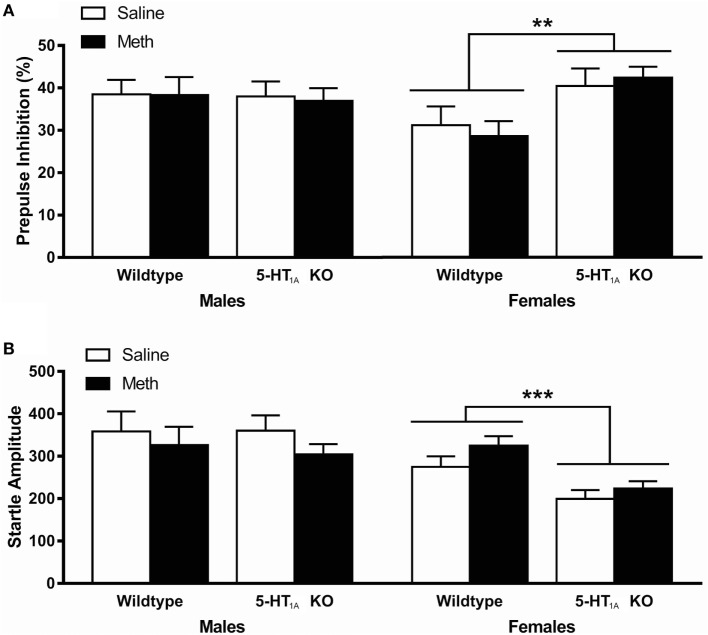
**(A)** Average prepulse inhibition (PPI) across all four PP intensities was significantly higher in 5-HT_1A_ knockout (KO) females compared to wild-type (WT) females. There was no difference in PPI between any groups in male mice. **(B)** Average startle responses across all four startle blocks were significantly lower in female 5-HT_1A_ KO mice compared to WT, and higher in female Meth pretreated compared to saline pretreated mice. There was no difference in startle between any groups in male mice. ** signifies genotype effect *p* < 0.01, *** signifies *p* < 0.001.

Analysis of PPI at 100 ms ISI showed a significant genotype × sex interaction [*F*_(1, 104)_ = 5.77, *p* = 0.018]; therefore, data were further analyzed separately for male and female mice (Figure 2A). Female, but not male, 5-HT_1A_ KO mice showed significantly higher PPI than WT mice [main effect of genotype *F*_(1, 54)_ = 9.08, *p* = 0.004]. However, there was no effect of Meth pretreatment on PPI in any of the groups.

Analysis of startle response showed that male mice had significantly higher startle compared to females [*F*_(1, 104)_ = 15.1, *p* < 0.001] while there was also a significant main effect of genotype [*F*_(1, 104)_ = 4.43, *p* = 0.038] and a genotype × sex interaction [*F*_(1, 104)_ = 4.52, *p* = 0.036] suggesting a genotype effect, which was dependent on sex (Figure 2B). We, therefore, again further analyzed results separately for male and female mice. As with PPI, male mice showed no difference between groups, while in females, 5-HT_1A_ KO mice showed significantly lower startle than WT mice [main effect of genotype *F*_(1, 54)_ = 17.6, *p* < 0.001]. It was also shown that female Meth pretreated mice had significantly higher startle compared to controls [main effect of pretreatment *F*_(1, 54)_ = 4.08, *p* = 0.048]; however, there was no genotype × pretreatment effect, suggesting that these results were independent of each other.

### Social Interaction

**Figure 3:**
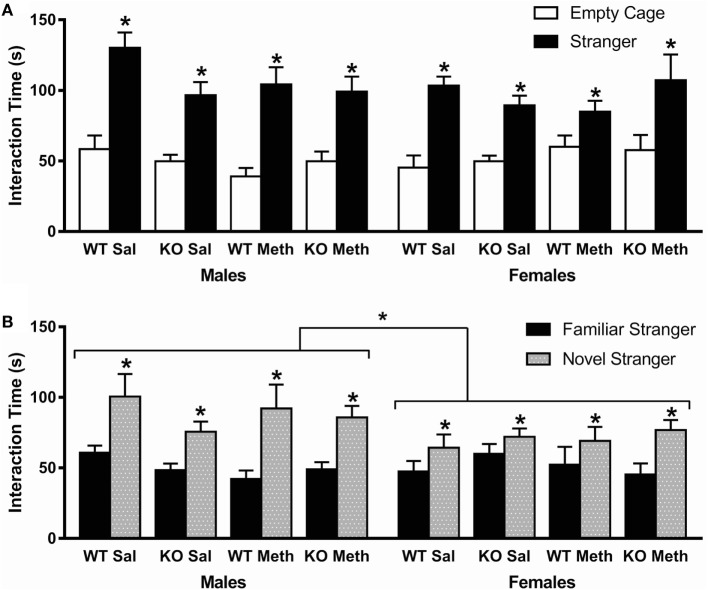
**Social behavior in the three-chamber task**. **(A)** Sociability, as measured by preference to interact with a stranger mouse compared to an empty cage, was normal in all groups (indicated by * above stranger). **(B)** Social novelty preference, as measured by preference to interact with novel stranger mouse, was also normal in all groups (indicated by * above stranger), although female mice showed a lower preference compared to males. *p < 0.05.

Analysis of total time spent interacting with the stranger mouse and empty cage showed a significant preference for the stranger mouse [*F*_(1, 78)_ = 150, *p* < 0.001], but there was no statistical interaction with genotype, pretreatment, or sex, indicating all groups showed similar sociability (Figure 3A).

In contrast, while analysis of the total time spent interacting with the familiar and novel stranger mice again showed a significant preference for the novel stranger [*F*_(1, 78)_ = 51.6, *p* < 0.001] and no statistical interaction with genotype or pretreatment, there was a significant interaction between stranger time and sex [*F*_(1, 78)_ = 5.6, *p* = 0.021; Figure 3B]. This suggests that there were no effects of genotype or Meth pretreatment on social novelty behavior, but that female mice show a decreased preference for the novel over the familiar stranger mouse.

### Elevated Plus Maze

**Figure 4:**
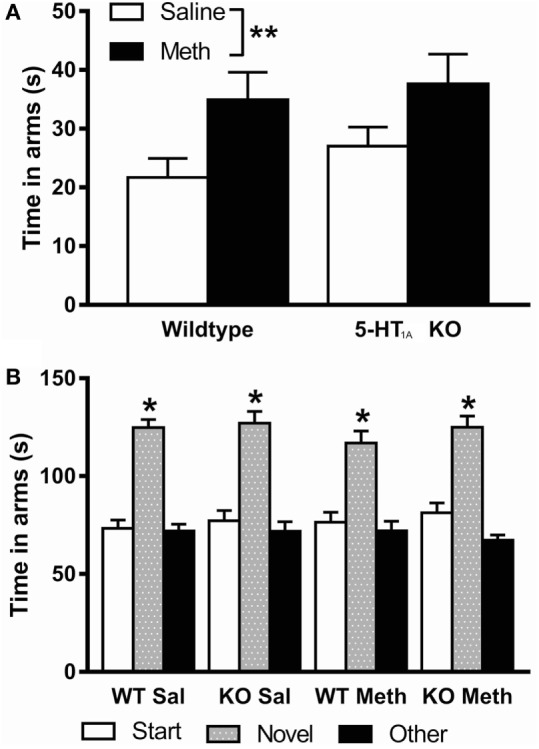
**(A)** Time spent in the open arms of the elevated plus maze was higher in Meth pretreated mice compared to saline pretreated. **(B)** Short-term spatial memory, measured by preference to spend time in the novel arm of the Y-maze, was normal in all groups (indicated by * above novel arm). *p < 0.05, **p < 0.01. Results are shown for males and females combined.

Analysis of time spent in the open arms of the EPM showed a significant main effect of Meth pretreatment [*F*_(1, 94)_ = 8.17, *p* = 0.005] reflecting that this pretreatment leads to increased time spent on the open arms of the EPM, suggesting decreased anxiety in these mice (Figure 4A). There was no significant main effect of genotype or sex; therefore, results have been presented for both sexes combined.

Total number of arm entries was analyzed as an indicator of activity on the plus maze (WT-Saline 42.2 ± 4.2, KO-Saline 45.1 ± 3.6, WT-Meth 46.5 ± 4.3, KO-Meth 47.0 ± 3.8). The results showed that there were no differences in activity between groups; therefore, overall activity did not have any effect on the different anxiety levels seen.

### Y-Maze

Analysis of total time spent in each arm of the Y-maze showed a significant main effect of arms [*F*_(2, 184)_ = 91.6, *p* < 0.001], but no significant interaction of time in arms with genotype, pretreatment, or sex indicating all groups showed a similar preference for the novel arm and have intact short-term spatial memory (Figure 4B). There was no significant main effect of sex; therefore, results have been presented for both sexes combined.

### Fear Conditioning

Analysis of context freezing (WT-Saline 42.3 ± 4.3, KO-Saline 45.6 ± 3.4, WT-Meth 44.7 ± 3.6, KO-Meth 40.9 ± 3.5%) and tone freezing (WT-Saline 43.5 ± 4.9, KO-Saline 55.5 ± 4.3, WT-Meth 49.0 ± 4.9, KO-Meth 48.1 ± 4.1) showed that there were no differences between the groups, suggesting that neither Meth pretreatment nor 5-HT_1A_ genotype have any effect on fear memory.

## Discussion

This study showed that, while both the Meth binge dosing and 5-HT_1A_ receptor KO genotype alter behavior in mice, 5-HT_1A_ KO mice did not respond differently to chronic Meth pretreatment in any of the behaviors tested. Meth pretreatment resulted in a heightened response to acute Meth and decreased levels of anxiety in both sexes, as well as increased startle responses in female mice only, independent of genotype. However, 5-HT_1A_ KO mice also showed an increased response to acute Meth in both sexes, as well as increased PPI and decreased startle response in female mice only, independent of Meth pretreatment. There were no effects on either short-term spatial memory in the Y-maze or conditioned fear memory induced by either Meth or 5-HT_1A_ receptor KO genotype.

These studies were first able to confirm that chronic escalating Meth exposure during adolescence/young adulthood leads to sensitization to the effects of an acute challenge dose of Meth in adulthood, consistent with previous studies (6). We were also able to confirm that 5-HT_1A_ KO mice have a greater response to acute Meth compared to WT consistent with their enhanced sensitivity seen following acute d-amphetamine previously (27). However, there was no statistical interaction between the two effects, as reflected by the observation that KO mice did not show an altered sensitivity to acute re-exposure to a single challenge dose of Meth compared to WT mice. These results suggest that the 5-HT_1A_ receptor is likely important in the acute action of Meth to induce hyperactivity but is not required for the development of sensitization induced by prior binge dosing of Meth. A previous study in the laboratory showed that the binding density of the dopamine transporter (DAT) and dopamine receptors D_1_ and D_2_ is only changed subtly in 5-HT_1A_ KO mice (27) and suggested that the interaction of 5-HT_1A_ receptors with dopamine release is at a more immediate functional level (45) rather that *via* more long-term changes in the expression of DAT or dopamine receptors.

Male mice showed no effect of genotype or Meth pretreatment on either PPI or startle response. Female 5-HT_1A_ KO mice, however, showed increased PPI and decreased startle responses compared to WT. The results are partly consistent with a previous study, which showed that administration of the 5-HT_1A_ antagonist, WAY100635, led to an increase in PPI in C57BL/6 mice (30); however, this was only done in male mice, which showed no changes in PPI between genotypes in the current study. While Meth pretreatment did not alter PPI in either sex, there was also a small but significant effect of Meth pretreatment to increase startle response in female mice; however, the effects of genotype and pretreatment were again independent of each other. Therefore, as with locomotor hyperactivity, Meth pretreatment had no specific effect in 5-HT_1A_ KO mice only. This is similar to previous studies using Meth pretreatment where no differences were seen in baseline PPI in either sex following binge Meth dosing (7).

There were no effects of Meth pretreatment or 5-HT_1A_ genotype on social behavior using the three-chamber sociability and social novelty preference tests. A previous study in the lab showed the same results for sociability, although suggested that Meth pretreatment decreased preference for social novelty to a lower level than saline pretreated mice in males only (8), while other studies have also shown impaired social behavior in animals previously exposed to Meth (46, 47).

5-HT_1A_ KO mice showed no signs of increased or decreased anxiety in the EPM, which is in contrast to other studies conducted in these mice, which have shown an anxious phenotype on the EPM and other tests of anxiety (22–26). Differences in experimental conditions such as conducting other behavioral tests prior to the elevated zero maze could contribute to the differences seen here compared to previous studies. Future studies should test for an anxiety phenotype in these mice prior to further behavioral testing to confirm presence of the phenotype. Importantly, however, Meth pretreatment led to increased time spent in the open arms of the plus maze, suggesting that these mice were less anxious than controls, but this effect was again independent of genotype. Previously, effects of chronic Meth pretreatment on anxiety have been variable. For example, a 10-day escalating dose protocol in mice induced no changes in anxiety measures in the EPM or light/dark box (48) while studies in rats showed increased anxiety or no change in the EPM depending on the age or extent of Meth exposure or time following exposure (49–51). These results suggest that dosing and testing protocol are very important for long-term effects of Meth on behavior.

A limitation of using 5-HT_1A_ KO mice is that the absence of these receptors during embryogenesis and development may influence the role of these receptors on behavior and the development of Meth sensitization. For example, changes in receptor density or function may change over time, and this could be altered by absence of the receptors during early development. Future studies could use conditional KO mice in which the receptor is deleted only in adulthood. Any differential role of presynaptic and postsynaptic 5-HT_1A_ receptors, which would both be absent in our KO mice, is also not addressed in this study. Previous research using 5-HT_1A_ agonists suggest that, while antagonists have no effect on behavioral sensitization (33, 34), the effects of the agonists may have been due to the activation of the presynaptic receptors preferentially over postsynaptic receptors (52). Studies show that both 8-OH-DPAT and osemozotan are able to prevent the development and expression of amphetamine or Meth-induced behavioral sensitization (35, 36), these effects were also reversed by the antagonist WAY100635, which has a greater affinity for presynaptic 5-HT_1A_ autoreceptors (53). Other limitations of this study are that the estrus cycle of female mice was not monitored, which could strengthen the results of any study using female animals, and the fact that we did not see an anxiety phenotype in the 5-HT_1A_ KO mice as discussed above.

While this study showed that there was no interaction between Meth pretreatment and the 5-HT_1A_ receptor in the protocols used, there is evidence that other 5-HT receptors may be involved in the long-term effects of Meth and Meth-induced sensitization. Repeated Meth administration failed to induce behavioral sensitization in 5-HT reuptake KO mice, but this was rescued by administration of a 5-HT_1B_ antagonist (54). Many different 5-HT receptor agonists and antagonists have been investigated for their role in behavioral sensitization induced by Meth. For example, the 5-HT_1B_ receptor antagonist, SB 216641, inhibited development but not expression of amphetamine-induced sensitization (55) while the 5-HT_2_ receptor antagonist, ritanserin, inhibited the development, expression, and maintenance of Meth-induced behavioral sensitization (56, 57). Taken together, the current findings in 5-HT_1A_ receptor KO mice do not support a role of these receptors in Meth-induced psychosis, although this does not rule out a role of other serotonin receptors, which can be addressed in future studies with selected KO lines such as 5-HT_1B_ and 5-HT_2_ receptor KO mice.

## Ethics Statement

All experimentation was approved by the La Trobe University Animal Ethics Committee and was conducted in accordance with the Australian Code of Practice for the Care and Use of Animals for Scientific Purposes set out by the National Health and Medical Research Council of Australia.

## Author Contributions

MB designed the study. EJ, DA, and TP performed all behavioral testing and collected data. EJ performed analysis and wrote the manuscript with the help of MB who edited the final version. All authors contributed to and approved the final version of this manuscript.

## Conflict of Interest Statement

The authors declare that the research was conducted in the absence of any commercial or financial relationships that could be construed as a potential conflict of interest.
